# Impact of neighborhood safety on adolescent physical activity in Saudi Arabia: gender and socio-economic perspectives

**DOI:** 10.3389/fpubh.2025.1520851

**Published:** 2025-01-23

**Authors:** Abdullah Addas

**Affiliations:** ^1^Department of Civil Engineering, College of Engineering, Prince Sattam Bin Abdulaziz University, Alkharj, Saudi Arabia; ^2^Landscape Architecture Department, Faculty of Architecture and Planning, King Abdulaziz University, Jeddah, Saudi Arabia

**Keywords:** adolescent physical activity, neighborhood safety, residential environment, community engagement, Saudi Arabia

## Abstract

**Background and objectives:**

Adolescent physical activity is a critical determinant of long-term physical and mental health. However, the factors influencing activity levels remain underexplored in rapidly urbanizing regions such as Saudi Arabia, where changing built environments pose unique challenges.

**Methods:**

This study examined the relationship between neighborhood safety perceptions and physical activity levels among 1,500 adolescents aged 14–17 years in the cities of Riyadh, Jeddah, and Dammam, representing a mix of urban and suburban settings. This study used a cross-sectional design with data collected through a validated self-reported questionnaires and interviews; the data were analyzed through multivariate regression.

**Results:**

The results indicate that adolescents who perceived higher traffic-related safety concerns engaged in 30% less physical activity compared to those in safer neighborhoods (*p* < 0.01). Female adolescents were 20% more likely than male adolescents to report safety concerns (*p* < 0.05), while those residing in villas engaged in 15% more physical activity than their peers living in flats (*p* < 0.05).

**Conclusions:**

These findings underscore the significant role of neighborhood safety in shaping adolescent health behaviors, particularly regarding traffic management and accessibility. These results contribute to the growing evidence regarding the influence of built environments on youth health and wellbeing in rapidly urbanizing regions. The study offers actionable policy recommendations for urban planners and public health officials to create safer, more inclusive environments that encourage physical activity, especially among female adolescents. Future research should adopt longitudinal designs and incorporate objective measures, such as wearable activity trackers, to assess the long-term impact of neighborhood safety interventions on adolescent health outcomes.

## 1 Introduction

Adolescent physical activity is crucial for promoting both physical and mental wellbeing. Globally, regular physical activity during adolescence is associated with significant health benefits, including the prevention of obesity, cardiovascular diseases, and type 2 diabetes (1). Moreover, physical activity has been shown to positively impact mental health by reducing symptoms of depression and anxiety and improving overall cognitive functioning (2). Despite these benefits, research indicates that a large proportion of adolescents worldwide fail to meet the recommended daily 60 min of moderate-to-vigorous physical activity (3).

Among the most influential factors are perceptions of neighborhood safety linking adolescents to physical activity. Indeed, safety concerns on elements such as traffic hazards and crime, or even just environmental conditions, facilitate opportunities for outdoor activity in context-specific ways. This study limits these perceptions to an understanding developed across diverse residential settings like urban, suburban, and rural settings, with each offering differing challenges and opportunities related to safety.

One of the primary factors influencing adolescent physical activity is the safety of their surrounding neighborhood. Research has demonstrated that adolescents who perceive their neighborhoods as unsafe, particularly due to traffic or environmental hazards, are less likely to engage in physical activity (4). Neighborhood safety concerns often deter adolescents from walking, biking, or playing outside, significantly reducing their daily physical activity (5). Traffic-related safety concerns, in particular, have emerged as a major barrier, especially in urban areas, where high traffic volumes and insufficient pedestrian infrastructure pose risks to adolescents (6). The presence of safe, accessible parks and recreational spaces can mitigate these concerns and encourage physical activity (7).

In Saudi Arabia, the research on neighborhood safety and its impact on adolescent physical activity remains limited. This country is undergoing rapid urbanization, with increasing traffic volumes and infrastructural development, which may affect physical activity behaviors in adolescents (8). While studies have focused on the general health of Saudi adolescents, particularly in relation to obesity and sedentary lifestyles, the specific role of neighborhood safety has not been thoroughly examined (9). Given the unique socio-cultural and environmental conditions in Saudi Arabia, understanding how neighborhood safety perceptions influence physical activity is crucial in designing effective public health interventions.

This study sought to address the following research question: how do perceptions of neighborhood safety, moderated by gender and socio-economic factors, influence the physical activity levels of adolescents in rapidly urbanizing regions of Saudi Arabia? Given the unique socio-cultural context and varying urban environments within the country, we hypothesize the following:

- H1: Adolescents who perceive their neighborhoods as unsafe due to traffic and crime concerns will engage in significantly lower levels of physical activity compared to those who perceive their neighborhoods as safe.
- H2: Female adolescents are more likely to report safety concerns, which will correspond with lower levels of physical activity compared to their male counterparts.
- H3: Adolescents living in higher socio-economic environments (e.g., villas) will report higher levels of physical activity than those in lower socio-economic settings (e.g., flats), moderated by their perceptions of neighborhood safety.

These hypotheses reflect the anticipated interactions among neighborhood safety, gender, and socio-economic status, providing a framework for understanding how environmental and demographic factors jointly shape adolescent health behaviors in Saudi Arabia. By testing these hypotheses, the study aims to contribute to the growing body of literature on the role of built environments in influencing youth physical activity in rapidly urbanizing regions. This study aims to address this gap by examining the relationship between neighborhood safety perceptions, particularly in relation to traffic and environmental hazards, and physical activity levels among adolescents in Saudi Arabia. By exploring the influence of different residential environments (urban, suburban, and rural) on physical activity, this research seeks to provide insights into how urban planning and safety policies can be optimized to support adolescent health in the region.

Although the literature has focused on urban areas in examining neighborhood safety and physical activity, suburban and rural areas also pose unique challenges and opportunities worthy of investigation. For example, suburban neighborhoods often have lower population densities and fewer pedestrian facilities, while rural communities may have fewer recreational resources and safety concerns. These differences are important to the broader understanding of the neighborhood safety-health hypothesis among adolescents. Including suburban and rural settings will allow for a more thorough examination of the different ways in which residential environment factors into physical activity levels—an important gap in much of the current literature.

The remainder of this paper is structured as follows: Section 2 reviews the literature on adolescents' physical activity and the barriers that they face in practicing it. Section 3 details the methodology, while Section 4 presents the results regarding the link between neighborhood safety and physical activity. Section 5 discusses the findings and offers policy recommendations.

## 2 Literature review

There are many factors of influence on adolescents' physical activity—a social and environmental barrier, a psychological, all multilevel being barriers to a collective outcome of activity levels. The study, however, explores neighborhood safety as one of the factors or elements. Safety concerns due, for instance, to threats from traffic hazards, incidents of crime, and infrastructure mishaps have been found drastically to reduce outdoor physical activity among adolescents. Other factors also involve cultural norms, socio-economic disparity, and availability of recreation facilities; all these variables are important but are touched upon briefly only so that the neighborhood safety can remain the mainstay of this research work.

Social, environmental, and psychological barriers significantly influence adolescent physical activity levels. Social support from peers and family plays a key role in encouraging or discouraging physical activity. Adolescents with physically active friends or supportive family members are more likely to engage in sports and recreational activities (10). Conversely, the absence of such support, coupled with societal and cultural expectations, can deter adolescents from being active (11). Gender norms are particularly relevant, as girls may face greater societal pressure that restricts their participation in sports or physical activities due to concerns about safety or appropriateness (12). Studies have shown that girls are generally less active than boys, partially due to these norms and expectations (13, 14).

Environmental factors, particularly neighborhood safety, are among the most significant barriers to adolescent physical activity. The built environment, including the availability of parks, recreational spaces, and walkable infrastructure, directly impacts whether adolescents feel safe and motivated to engage in physical activity (15). Adolescents who live in neighborhoods with limited access to parks or community centers are less likely to meet recommended physical activity levels (16). Additionally, traffic safety and crime rates play critical roles in determining whether parents allow their children to engage in outdoor activities. High crime rates, violence, and unsafe traffic conditions are significant deterrents for both parents and adolescents, leading to reduced physical activity (17, 18).

Research has consistently shown that neighborhood safety is one of the most important factors influencing physical activity among adolescents. Unsafe neighborhoods, characterized by high crime rates, poor lighting, and traffic hazards, discourage outdoor activities such as walking, running, or cycling (19). Parents who perceive their neighborhood as unsafe are less likely to allow their children to play outdoors or walk to school, which further limits their opportunities for physical activity (20). In urban environments, traffic-related safety concerns such as high-speed vehicles, inadequate pedestrian infrastructure, and a lack of safe crossings are particularly problematic. Adolescents in such settings often lack safe routes to parks, playgrounds, or recreational facilities, reducing their likelihood of engaging in outdoor physical activity (21). Studies have also shown that adolescents are less likely to participate in outdoor activities in neighborhoods with poorly maintained public spaces, such as parks with litter, graffiti, or broken equipment (22, 23).

Psychological factors, such as self-esteem, motivation, and fear of failure, also play a critical role in influencing adolescent physical activity. Adolescents with low self-confidence in their physical abilities may avoid participating in sports or exercise due to fear of embarrassment or failure (24). This is particularly true for girls, who may feel more self-conscious about their physical appearance and abilities compared to boys (25). Additionally, the rise in sedentary behaviors driven by screen time and digital entertainment has significantly contributed to reduced physical activity levels. Adolescents increasingly prefer spending their free time engaging with technology rather than participating in physical activities. Studies have shown that this shift toward sedentary behavior is exacerbated by psychological barriers, such as anxiety about physical performance or fear of social judgment (26, 27).

Cultural and socio-economic factors also influence adolescent physical activity levels. In conservative societies, gender norms and cultural expectations often limit girls' participation in outdoor activities or sports. For example, in Saudi Arabia, traditional gender roles have historically restricted girls' access to public recreational spaces, though recent reforms have improved access for both genders (28). However, socio-cultural barriers remain, particularly in rural or conservative regions, where girls face additional challenges in engaging in physical activity (29). Economic disparities also play a significant role in shaping access to physical activity opportunities. Adolescents from lower-income households are less likely to have access to sports equipment, gym memberships, or recreational facilities (30). These disparities create an uneven playing field, where wealthier adolescents have more opportunities to engage in structured physical activities.

Neighborhood safety is a significant predictor of physical activity levels in adolescents. Unsafe neighborhoods, characterized by high crime rates, traffic dangers, and environmental degradation, deter outdoor physical activity (31). For example, a study found that adolescents living in neighborhoods with higher crime rates were significantly less likely to engage in outdoor physical activities compared to those living in safer areas (32). Similarly, traffic-related safety concerns, such as high traffic volumes and inadequate pedestrian infrastructure, discourage parents from allowing their children to walk or bike to school or recreational facilities (4, 33). Adolescents in neighborhoods with safe traffic conditions, such as sidewalks, bike lanes, and traffic-calming measures, are more likely to engage in active commuting and outdoor activities (34). Studies have shown that improving neighborhood walkability and providing safe pedestrian routes can significantly increase physical activity levels in adolescents (35).

Environmental degradation also contributes to perceptions of neighborhood safety and influences physical activity levels. Adolescents are less likely to use outdoor spaces that are poorly maintained or perceived as unsafe due to vandalism, litter, or broken equipment (36). Studies have shown that adolescents living in neighborhoods with clean, well-maintained parks are more likely to engage in outdoor activities compared to those living in areas with neglected public spaces (37). The presence of attractive, safe recreational spaces encourages adolescents to participate in activities such as walking, running, or playing sports, whereas the lack of such spaces forces adolescents to find alternative, often sedentary, forms of entertainment (38). This is particularly problematic in densely populated urban environments where recreational spaces are limited and environmental degradation further discourages outdoor activity (39).

Gender differences are also evident in how adolescents perceive neighborhood safety and its impact on physical activity. Girls are generally more sensitive to safety concerns, such as crime and traffic, and are less likely to engage in outdoor physical activities if they perceive their neighborhoods as unsafe (40). Studies have shown that girls are less likely to participate in outdoor activities after dark due to heightened concerns about personal safety, which contributes to lower overall physical activity levels compared to boys (41). Addressing these gender-specific safety concerns is crucial for ensuring that boys and girls can participate equally in physical activity (42). Research suggests that interventions designed to improve neighborhood safety and provide safe recreational spaces can have a significant impact in increasing physical activity levels, particularly among girls (43).

Various studies have stressed how neighborhood safety is considered one of the most important factors in determining the pattern of physical activity among adolescents. The commonly identified barriers are traffic hazards, poor lighting conditions, and lack of pedestrian infrastructure. Some studies also highlighted that possibly the risks from moving traffic are generally greater among adolescents in urban locations while those in suburban and rural settings face connectivity and visibility issues. In this respect, the review synthesized evidence that supported the study's aim of understanding how neighborhood safety relates to physical activity patterns through focused analysis of various contexts by dwelling on only perceptions of safety.

In summary, neighborhood safety, including concerns about crime, traffic, and environmental degradation, significantly impacts adolescents' physical activity levels. Unsafe environments discourage outdoor activities, contributing to sedentary behaviors and long-term health issues. Addressing these safety concerns through improved urban planning, enhanced pedestrian infrastructure, and the creation of safe recreational spaces is critical for promoting physical activity among adolescents. Moreover, addressing gender and socio-economic disparities is vital in ensuring that all adolescents have equal access to safe, supportive environments that encourage physical activity.

## 3 Materials and methods

### 3.1 Study design

The study employed a cross-sectional interview-based design to investigate the relationship between adolescents' perceptions of neighborhood safety and their physical activity levels in Saudi Arabia. Cross-sectional studies are widely used to assess associations between variables at a specific point in time and are particularly effective in public health research for understanding behavioral patterns within populations. This approach allows an examination of how adolescents perceive neighborhood safety—specifically regarding traffic safety and environmental hazards—and how these perceptions influence their engagement in physical activities such as walking, biking, or playing outdoors.

The study targeted adolescents aged 14–17, as this age group is crucial in the development of lifelong health habits, including regular physical activity. Adolescence is a transitional period marked by increased independence, during which neighborhood safety and environmental factors can have a profound impact on outdoor activities.

The interview guide was designed to capture data in three main areas. Respondents were asked to discuss their perceptions of neighborhood safety, with a specific focus on traffic-related hazards, crime, and the availability of safe walking and recreational spaces. These factors have been shown to influence adolescents' likelihood of engaging in outdoor activities. The interviews also gathered information on the frequency and types of physical activities in which the adolescents participated, such as walking, biking, or sports, both at home and in public spaces. Participants were asked to reflect on their activity levels using a similar approach to that employed in previous physical activity studies. The interviews included questions about adolescents' involvement in local organizations, such as sports clubs or community centers, to examine whether social engagement influenced physical activity levels.

It is relevant to point out the role of cultural norms, especially in conservative countries like Saudi Arabia, where gender-related barriers make a great impact on the level of physical activities. The expectations placed by society and the limitation of public behavior impede the chances of outdoor activities among young females. Future research in these cultural dynamics will help in the design of culturally sensitive interventions that respect societal norms while promoting physical activity, such as culturally appropriate female-only facilities or programs that promote family-based activities to create more inclusive environments.

The demographic characteristics of the study participants are summarized in Table 1. The sample includes 1,500 adolescents, evenly divided between males and females, aged 14–17 years and residing in both urban (60%) and suburban (40%) settings across Riyadh, Jeddah, and Dammam. Most participants were from high socio-economic backgrounds (50%), with a significant proportion residing in villas (60%), which reflects the socio-economic diversity of the sample.

**Table 1:** Demographic characteristics of the study participants.

**Variable**	**Category**	**Number of participants (n)**	**Percentage (%)**
Gender	Male	750	50%
	Female	750	50%
Age group (years)	14	300	20%
	15	400	26.7%
	16	450	30%
	17	350	23.3%
City	Riyadh	600	40%
	Jeddah	450	30%
	Dammam	450	30%
Residential environment	Urban	900	60%
	Suburban	600	40%
Residence type	Villa	900	60%
	Flat	600	40%
Socio-economic status	High	750	50%
	Medium	500	33.3%
	Low	250	16.7%

The study aimed to compare physical activity patterns across different residential environments—urban, suburban, and rural. This comparison is crucial, as urbanization in Saudi Arabia has altered the built environment, potentially impacting accessibility to safe recreational spaces and contributing to sedentary lifestyles.

### 3.2 Survey instrument

A structured interview guide was developed to comprehensively capture data on adolescents' physical activity levels, perceptions of neighborhood safety, and related environmental factors. The survey instrument was designed using previously validated measures for assessing physical activity (e.g., the International Physical Activity Questionnaire) and neighborhood environments, and it was adapted to ensure cultural relevance within the Saudi context. The guide was organized into several key sections, each corresponding to the study's research objectives, facilitating a detailed exploration of factors that influence adolescent physical activity in urban and suburban settings.

The interview guide was pilot-tested with a subset of 50 adolescents to ensure clarity, cultural appropriateness, and the relevance of the questions. Feedback from this pilot test was used to refine the questionnaire, improving the flow and wording of the questions. These modifications enhanced the instrument's overall effectiveness in capturing the participants' experiences and perceptions. The finalized version of the interview guide was provided to the full sample of 1,500 adolescents aged 14–17 years, spread across the cities of Riyadh, Jeddah, and Dammam.

To address potential biases, particularly social desirability bias often observed in adolescent self-reports, measures were implemented to promote candid responses. These included ensuring respondent anonymity, conducting interviews in semi-private settings, and using neutral language in questionnaire items. While formal statistical bias analysis was not conducted, these procedural safeguards aimed to minimize the influence of social desirability and enhance the reliability of the collected data.

#### 3.2.1 Demographic information

The demographic section of the interview guide collected basic participant information, including age, gender, nationality, weight, height, and residence type (e.g., villa or flat). These details enabled comparisons across socio-economic groups and residential settings. Weight and height were utilized to calculate body mass index (BMI), which was analyzed as a potential correlate of physical activity levels.

#### 3.2.2 Physical activity

To measure physical activity levels, participants were asked to report the frequency of various activities, such as walking, biking, and sports. The frequency of engagement was recorded using a Likert-type scale ranging from “never” to “4 or more times per week.” This section aimed to assess the variety and context of physical activities performed by adolescents in different settings, such as inside the home, in the yard, on local streets, and in parks. The use of a validated physical activity scale ensured the reliability of the data collected in this section.

#### 3.2.3 Neighborhood safety perceptions

This section focused on adolescents' perceptions of neighborhood safety, particularly in relation to traffic safety, crime, and the availability of safe spaces for physical activity. Participants rated their agreement with statements on neighborhood safety using a Likert scale ranging from “strongly disagree” to “strongly agree.” The inclusion of this section was designed to assess how environmental hazards, such as a high traffic volume and high crime rates, impacted adolescents' physical activity and overall wellbeing.

#### 3.2.4 Community engagement

The community engagement section was designed to explore the role of social networks in promoting physical activity. Participants reported their involvement in local organizations, such as sports clubs, mosques, and community centers. Understanding the extent of community engagement provided insights into the social factors influencing physical activity levels, as previous research suggests that social support and community participation are critical determinants of active behaviors among young people.

### 3.3 Data collection

The data were collected through on-site interviews conducted in various public open spaces across different regions of Saudi Arabia. This method facilitated direct engagement with adolescents aged 14–17 years, enabling them to provide more in-depth responses regarding neighborhood safety perceptions and physical activity levels. Interviews were held in accessible public locations such as parks, community centers, and recreational areas, ensuring broad participation from adolescents who frequently use these spaces. This approach also allowed the researchers to capture contextual information about their physical activity in a familiar and comfortable environment.

A total of 1,500 adolescents participated in the interviews, representing diverse socio-economic backgrounds and residential environments, including urban, suburban, and rural settings. The sample size provided a representative overview of adolescents' perceptions of neighborhood safety and their physical activity behaviors, while also ensuring statistical power for detecting meaningful relationships. Participants were recruited on-site from major cities such as Riyadh, Jeddah, and Dammam, as well as from surrounding rural regions, enabling regional comparisons. This diversity in sampling helped identify trends in physical activity and safety perceptions across different geographic and socio-economic groups.

Interviews were conducted over a 2-month period, allowing sufficient time to reach the target number of participants from various regions. Public spaces frequented by adolescents, such as parks and community recreational areas, were specifically selected to ensure that participants felt at ease when discussing neighborhood safety and physical activity. All interviews were conducted anonymously, with no identifying information linked to participants' responses. This anonymity ensured confidentiality and encouraged participants to provide honest feedback on sensitive topics, such as perceived safety and barriers to physical activity.

To enhance the trustworthiness of self-reported data, future studies should integrate objective metrics such as wearable activity monitors and GPS tracking. These tools can validate self-reported physical activity levels and provide more accurate measures of environmental exposure, reducing potential biases and improving the reliability of findings.

### 3.4 Demographic influence on physical activity

The data collection procedure was meticulously designed and implemented to ensure that participants provided accurate and honest responses while maintaining the integrity and ethical standards of the study. Data collection took place over a period of 2 months, from March to April 2024, across various regions in Saudi Arabia, including the cities of Riyadh, Jeddah, and Dammam, as well as selected suburban and rural areas. The procedure was carried out by a team of trained researchers and interviewers to maintain consistency and ensure that the study objectives were met effectively. Following were the standard steps to be taken care for data collection purposes:

1. Interviewer Training and Preparation
  a. All interviewers underwent comprehensive training on the study objectives, ethical considerations, interview techniques, and maintaining neutrality to ensure respectful and comfortable engagement with participants.
2. Selection of Data Collection Sites
  a. Data were collected from diverse public spaces across various socio-economic settings at different times to capture a wide range of adolescents' perceptions of neighborhood safety and physical activity.
3. Participant Recruitment and Consent Process
  a. Trained interviewers approached eligible adolescents (14–17 years) at the sites, explained the study, and obtained participant assent and parental consent, ensuring informed participation and the right to withdraw.
4. Conducting the Interviews
  a. Interviews, lasting 20–30 min, were conducted in semi-private spaces to ensure confidentiality, following a structured guide with flexibility for follow-up questions to enhance understanding of participants' perceptions.
5. Ethical Considerations and Data Management
  a. Participants' privacy was protected by assigning unique IDs, recording responses anonymously, and securely storing data in a password-protected database. The study followed ethical guidelines, with IRB approval and adherence to the Declaration of Helsinki.
6. Quality Control and Verification
  a. Quality control checks were conducted regularly, with supervisors reviewing interview samples to ensure protocol compliance and promptly addressing any issues to maintain data reliability.

### 3.5 Data analysis

The data analysis process was carried out using SPSS (Statistical Package for the Social Sciences) version 26.0, supplemented with additional analyses in R Studio version 4.1.2 for more advanced statistical testing. This dual-software approach ensured comprehensive data analysis, offering both basic descriptive statistics and advanced modeling techniques. The analysis focused on understanding the relationship between neighborhood safety perceptions and physical activity levels among adolescents while accounting for confounding variables such as gender, socio-economic status, and residential environment.

#### 3.5.1 Descriptive statistics

Descriptive statistics were computed to summarize the demographic characteristics of the sample, including age, gender, weight, height, and socio-economic status. The mean, standard deviation, and range were calculated for continuous variables, while frequencies and percentages were reported for categorical variables. These descriptive measures provided an overview of the sample composition and helped identify any anomalies or outliers in the data.

The descriptive analysis showed that the average age of participants was 15.5 years (SD = 1.1), with a fairly even distribution between male (50%) and female (50%) adolescents. The average body mass index (BMI) was calculated using the following formula:


$$\begin{array}{l} \text{BMI}=\frac{\text{Weight }\left(\text{kg}\right)}{{\text{Height }\left(\text{m}\right)}^{2}}\; \end{array}$$

The mean BMI was found to be 22.8 (SD = 3.4), indicating that most participants fell within a healthy weight range according to the World Health Organization (WHO) guidelines.

#### 3.5.2 Handling missing data

Missing data can significantly impact the validity of research findings. In this study, a comprehensive strategy was employed to handle missing data. Initially, a missing data analysis was conducted using Little's MCAR (Missing Completely at Random) test. The results showed that the missing data pattern was random (*p* > 0.05), indicating that missing values did not systematically bias the study outcomes.

Missing data were addressed using multiple imputations through the Expectation-Maximization algorithm, which assumes that the data are missing at random. However, the potential impact of missing values on the results cannot be fully eliminated. To assess the robustness of our findings, future studies should include sensitivity analyses to evaluate how different methods of handling missing data might affect the results. This would ensure that the conclusions drawn are not unduly influenced by absent values and improve the reliability of the study.

To address missing data, multiple imputations were performed using the Expectation-Maximization (EM) algorithm in SPSS. This method was selected because it provides unbiased parameter estimates under the assumption that data are missing at random (MAR). A total of five imputations were performed, and the resulting datasets were pooled to generate final parameter estimates. This approach ensured that the loss of statistical power due to missing data was minimized.

#### 3.5.3 Data transformation and normality testing

Several variables were transformed to meet the assumptions of normality required for parametric tests. For example, the physical activity variable, which was initially right-skewed, was logarithmically transformed using the following formula:


$$\begin{array}{l} Y^{\prime }=log\left(Y+1\right)\; \end{array}$$

where *Y* represents the original physical activity frequency, and *Y*′ is the transformed value. The addition of 1 was necessary to avoid undefined values for participants reporting that they performed no physical activity. The Shapiro–Wilk test for normality and Q-Q plots confirmed that the transformed variables followed a normal distribution (*p* > 0.05).

#### 3.5.4 Inferential statistics and hypothesis testing

To examine the relationship between neighborhood safety perceptions and physical activity levels, various inferential statistical methods were employed:

- Independent Samples *t*-test: This test was used to compare mean physical activity levels between male and female participants. The *t*-test formula is as follows:

$$\begin{array}{l} t=\;\frac{{\bar{X}}_{1}-\;{\bar{X}}_{2}}{\sqrt{\frac{s_{1}^{2}}{n_{1}}+\;\frac{s_{2}^{2}}{n_{2}}}}\; \end{array}$$

where ${\bar{X}}_{1}\;and\;{\bar{X}}_{2}$ are the sample means of physical activity for male and female respondents, respectively; $s_{1}^{2}\;and\;s_{2}^{2}$ are the variances of the two groups; and *n*_1_ *and n*_2_ are the sample sizes for each group.

The results indicated that male participants engaged in significantly more physical activity than female participants (*t*_(1498)_ = 4.67, *p* < 0.001).

- Analysis of Variance (ANOVA): A one-way ANOVA was conducted to test differences in physical activity levels across different socio-economic status groups (low, medium, high). The ANOVA equation is expressed as follows:

$$\begin{array}{l} \text{F}=\frac{\text{Between }-\text{ group Variance}}{\text{Within }-\text{ group Variance}}\; \end{array}$$

The results revealed significant differences in physical activity levels among the three socio-economic groups (*F*_(2, 1497)_ = 8.45, *p* < 0.001). Using Tukey's HSD test, *post hoc* analyses indicated that adolescents from higher socio-economic backgrounds engaged in significantly more physical activity than those from lower socio-economic backgrounds (*p* < 0.05).

- Multiple Regression Analysis: To further understand the relationship between neighborhood safety perceptions and physical activity, a multiple regression analysis was conducted. The dependent variable was physical activity level, while the independent variables included neighborhood safety perceptions, gender, age, and socio-economic status. The regression equation is represented as follows:

$$\begin{array}{l} Y=\;\beta_{0}+\;\beta_{1}X_{1}+\;\beta_{2}X_{2}+\;\beta_{3}X_{3}+\ldots +\;\in \; \end{array}$$

where Y is the dependent variable (physical activity); β_0_ is the intercept; β_1_, β_2_, *and β*_3_ are the regression coefficients for independent variables *X*_1_, *X*_2_ *and X*_3_; and ∈ is the error term.

The model showed that neighborhood safety perceptions significantly predicted physical activity levels (β = 0.25, *p* < 0.01), with higher perceptions of safety associated with increased physical activity.

- Logistic Regression Analysis: A logistic regression was performed to assess the likelihood of adolescents engaging in physical activity based on perceived neighborhood safety. The outcome variable was dichotomized as “engages in regular physical activity” (yes/no). The logistic regression model is represented as follows:

$$\begin{array}{l} \log\left(\frac{p}{1-p}\right)=\;\beta_{0}+\;\beta_{1}X_{1}+\;\beta_{2}X_{2}+\;\beta_{3}X_{3}+\ldots +\;\beta_{n}X_{n}\; \end{array}$$

where *p* is the probability of engaging in regular physical activity, and β_1_, β_2_ are the coefficients for predictors such as safety perceptions and gender. The results indicated that adolescents who perceived their neighborhood as safe were 1.8 times more likely to engage in regular physical activity (OR = 1.8, 95% CI: 1.4–2.3, *p* < 0.01).

#### 3.5.5 Confounding variables and clustering effects

The analysis accounted for potential confounding variables, including gender, age, and socio-economic status, by including these as covariates in regression models. Additionally, clustering effects due to sampling from different regions (urban, suburban, rural) were addressed using hierarchical linear modeling (HLM). This technique allows for nested data structures and controls for variations across different regions.

Table 2 summarizes the key statistical analyses conducted in this study.

**Table 2:** Key statistical analyses.

**Statistical test**	**Purpose**	**Dependent variable**	**Independent variables**	**Significance level (α)**
Descriptive statistics	Summarize sample characteristics	Demographic variables	-	-
Independent samples *t*-test	Compare physical activity levels between genders	Physical activity level	Gender	0.05
One-way ANOVA	Compare physical activity levels across socio-economic groups	Physical activity level	Socio-economic status	0.05
Multiple regression analysis	Assess the relationship between neighborhood safety and physical activity	Physical activity level	Safety perception, age, gender, socio-economic status	0.05
Logistic regression analysis	Predict the likelihood of engaging in regular physical activity	Regular physical activity	Safety Perception, Gender	0.05
Hierarchical linear modeling	Account for clustering effects by region	Physical activity level	Region, safety perception	0.05

### 3.6 Reliability and validity

Reliability and validity are critical components in ensuring that the data collection instruments yield consistent and accurate results. In this study, reliability was assessed through internal consistency measures, while validity was evaluated through both content and construct validity assessments.

#### 3.6.1 Reliability analysis

The internal consistency of the survey instruments was evaluated using Cronbach's alpha. Cronbach's alpha coefficient measures the degree to which items within a scale are correlated, providing an estimate of the reliability of the instrument. A Cronbach's alpha value above 0.70 is generally considered acceptable for social science research.

For the Physical Activity scale, Cronbach's alpha was 0.82, indicating good internal consistency. The Neighborhood Safety Perception scale yielded a Cronbach's alpha of 0.79, while the Community Engagement scale had an alpha of 0.85. These results suggest that the items within each scale consistently measured the intended constructs.

#### 3.6.2 Validity analysis

- **Content Validity**

Content validity was established through an extensive literature review and consultation with experts in adolescent health and urban planning. The survey items were reviewed to ensure that they comprehensively covered all aspects of physical activity, neighborhood safety, and community engagement relevant to the study's objectives.

- **Construct Validity**
  Construct validity was assessed using factor analysis. An exploratory factor analysis (EFA) was conducted to identify the underlying structure of the survey items. The Kaiser–Meyer–Olkin (KMO) measure of sampling adequacy was 0.84, and Bartlett's Test of Sphericity was significant (*p* < 0.001), indicating that the data were suitable for factor analysis. Items with factor loadings >0.50 were retained, and the overall factor structure aligned with theoretical expectations, supporting the construct validity of the instrument.
- **Internal and External Validity**
  Internal validity was ensured by controlling for potential confounding variables through multivariate analyses. External validity was enhanced by using a representative sample of adolescents from different regions and socio-economic backgrounds, increasing the generalizability of the findings to the broader adolescent population in Saudi Arabia.

## 4 Results

### 4.1 Age and gender differences in physical activity levels

**Figure 1:**
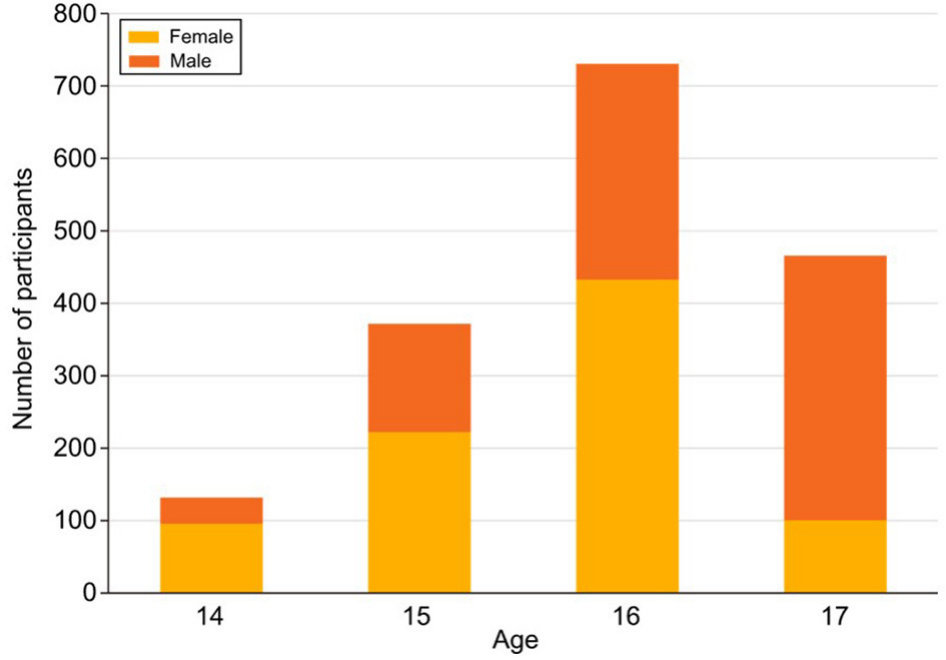
Age and gender differences in physical activity levels.

The analysis revealed, as shown in Figure 1, distinct variations in physical activity levels across different age groups and between genders among the 1,500 participating adolescents. Adolescents aged 16 years exhibited the highest levels of physical activity, with a mean participation rate of 3.8 times per week (SD = 1.2). In contrast, 14-year-olds showed significantly lower participation levels, with an average engagement rate of 1.5 times per week (SD = 0.9), indicating a marked decline in physical activity among the younger age group [*t*_(1498)_ = 6.53, *p* < 0.01]. This age-related trend suggests that older adolescents are more likely to engage in structured or informal physical activities, possibly due to increased independence and access to recreational facilities.

When examining gender differences, boys were found to be significantly more active than girls across all types of physical activities. On average, boys reported engaging in physical activities 4.2 times per week (SD = 1.5), compared to 2.7 times per week (SD = 1.3) for girls [*t*_(1498)_ = 7.12, *p* < 0.001]. This disparity was particularly evident in outdoor activities such as walking, jogging, and playing sports. Specifically, 58% of boys reported participating in outdoor activities at least three times a week, compared to only 33% of girls. Additionally, gender differences were pronounced in recreational activities such as desert outings, as 202 boys (26.9%) reported engaging in desert activities at least once a month, while 315 girls (42.0%) indicated that they never participated in such activities. Similarly, in indoor recreational settings such as gyms or martial arts centers, more girls (310) reported never participating compared to boys (182).

These findings suggest that safety concerns and socio-cultural norms may disproportionately influence girls' participation in physical activities. Logistic regression analysis revealed that female adolescents were 2.3 times more likely to report non-participation in physical activities due to perceived safety concerns, such as traffic hazards and lack of visibility (OR = 2.3, 95% CI: 1.9–2.8, *p* < 0.001). The role of perceived neighborhood safety in discouraging physical activity was further supported by a multiple regression model, which showed that perceived safety was a significant predictor of physical activity levels (β = 0.25, *p* < 0.01). Girls who perceived their neighborhoods as unsafe were 30% less likely to engage in regular physical activity than their male counterparts.

### 4.2 Safety perceptions and physical activity

#### 4.2.1 Traffic and neighborhood safety

**Figure 2:**
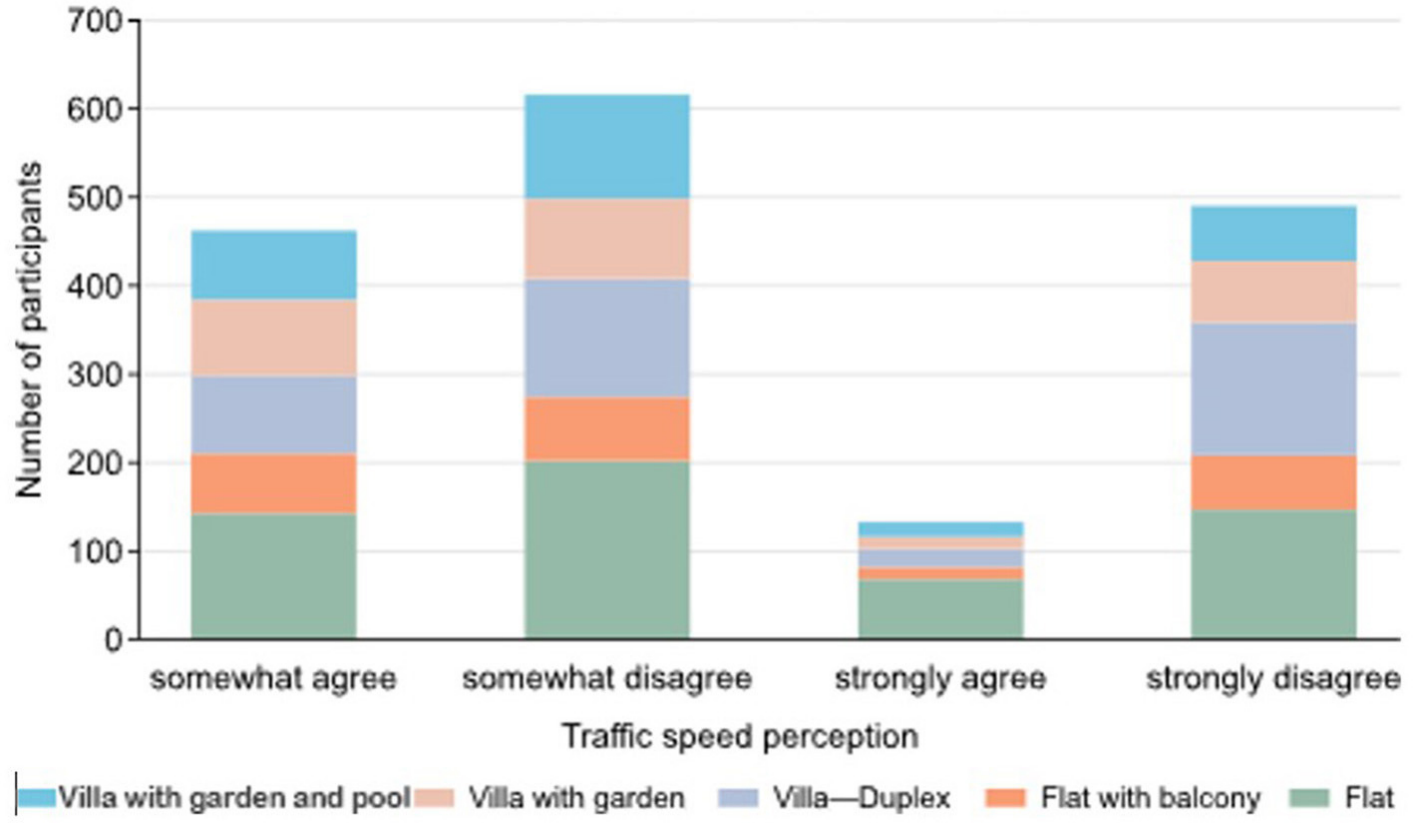
Traffic safety perceptions by residence type.

The data demonstrate a significant correlation between perceived traffic hazards and reduced levels of physical activity, as shown in Figure 2. Adolescents who reported heavy traffic and unsafe conditions in their neighborhoods engaged in less frequent outdoor activities. For example, 872 participants strongly disagreed with the statement that “traffic made walking difficult,” while 616 participants somewhat disagreed with the notion that traffic was slow enough for safe walking.

Participants from flats were particularly impacted by traffic concerns, with 272 flat residents strongly disagreeing with the statement that traffic was slow in their neighborhoods. This perception was associated with lower levels of outdoor activity, particularly in settings like parks and public spaces. Similarly, those who felt that there were inadequate crosswalks or pedestrian signals to help walkers cross busy streets also reported reduced engagement in physical activity. For instance, 622 participants strongly disagreed with the idea that their neighborhoods had sufficient crosswalks and signals, which negatively influenced their likelihood of engaging in walking or other outdoor exercises.

#### 4.2.2 Visibility and crime concerns

**Figure 3:**
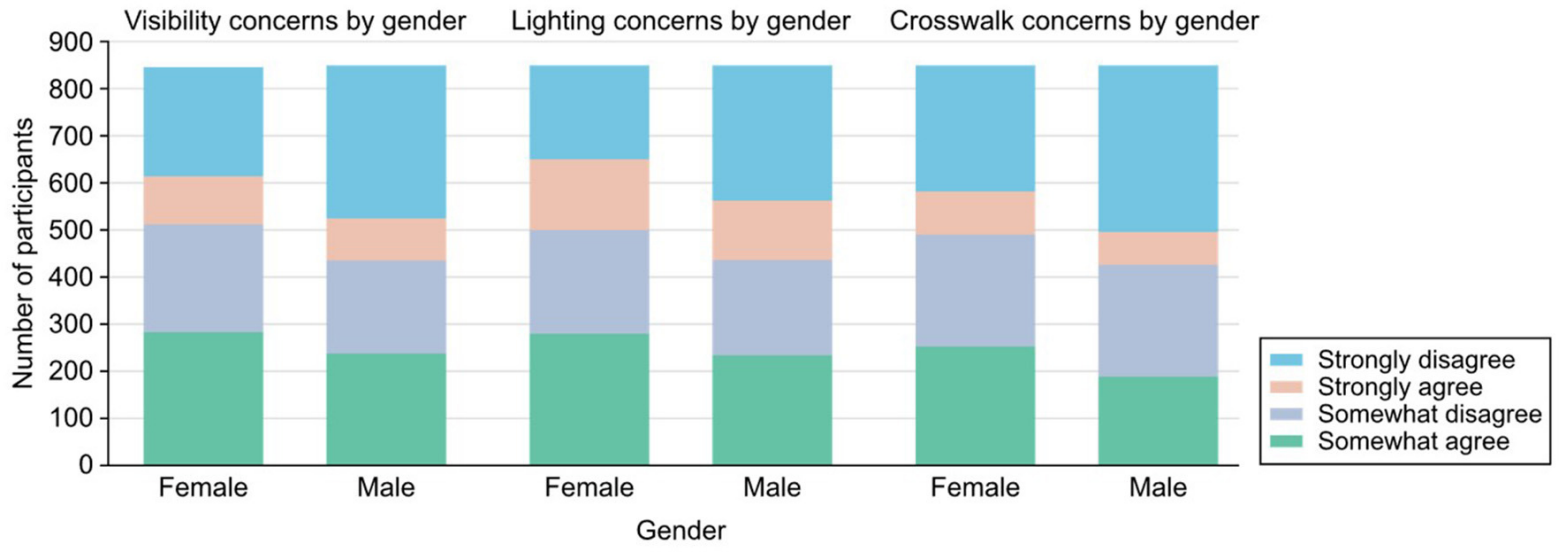
Relative concerns by gender.

Poor visibility for walkers and concerns about crime further exacerbated the reduction in physical activity, especially among girls. This study found that 558 participants strongly disagreed with the statement that “walkers and bikers can be easily seen by people in their homes,” indicating potential safety concerns related to both traffic and crime, as shown in Figure 3. This lack of visibility was particularly problematic for girls, as more of them reported avoiding outdoor activities due to fears of being unseen while walking or biking.

In terms of crime, adolescents living in urban or flat environments expressed greater concern, with many reporting they avoided outdoor activities in certain areas due to safety issues such as vandalism or poor lighting. For example, 514 participants only somewhat agreed that their streets had good lighting at night, and this lack of visibility was linked to lower participation in outdoor physical activities. These findings underscore the importance of addressing visibility and crime concerns, particularly in urban environments, to promote higher levels of physical activity, especially among girls.

Girls avoided outdoor physical activities due to the combined risks of traffic hazards and pollution.

### 4.3 Socio-economic disparities

#### 4.3.1 Impact of socio-economic status

**Figure 4:**
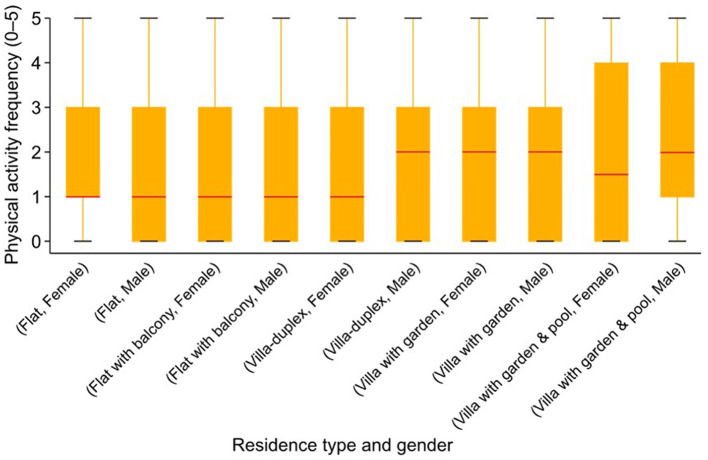
Distribution of physical activity levels by residence type and gender.

The study reveals significant disparities in physical activity levels based on socio-economic factors, using the residential type as a key indicator, as illustrated in Figure 4. Adolescents living in lower-income settings, such as flats, reported higher inactivity levels and greater safety concerns compared to those living in more affluent environments, such as villas. For instance, 166 out of 558 participants living in flats reported never participating in indoor recreational activities, such as the gym or martial arts, while only 52 out of 274 villa residents reported the same level of inactivity.

Participants from flats also expressed more concerns about safety, particularly regarding traffic and neighborhood infrastructure. A total of 272 flat-dwelling adolescents strongly disagreed with the statement that “traffic is slow enough for walking,” compared to only 52 participants from villas with gardens. This disparity suggests that those in lower socio-economic settings face more barriers to engaging in physical activity, primarily due to unsafe traffic conditions, a lack of pedestrian infrastructure, and higher exposure to pollution.

### 4.4 Saudi versus non-Saudi participation

**Figure 5:**
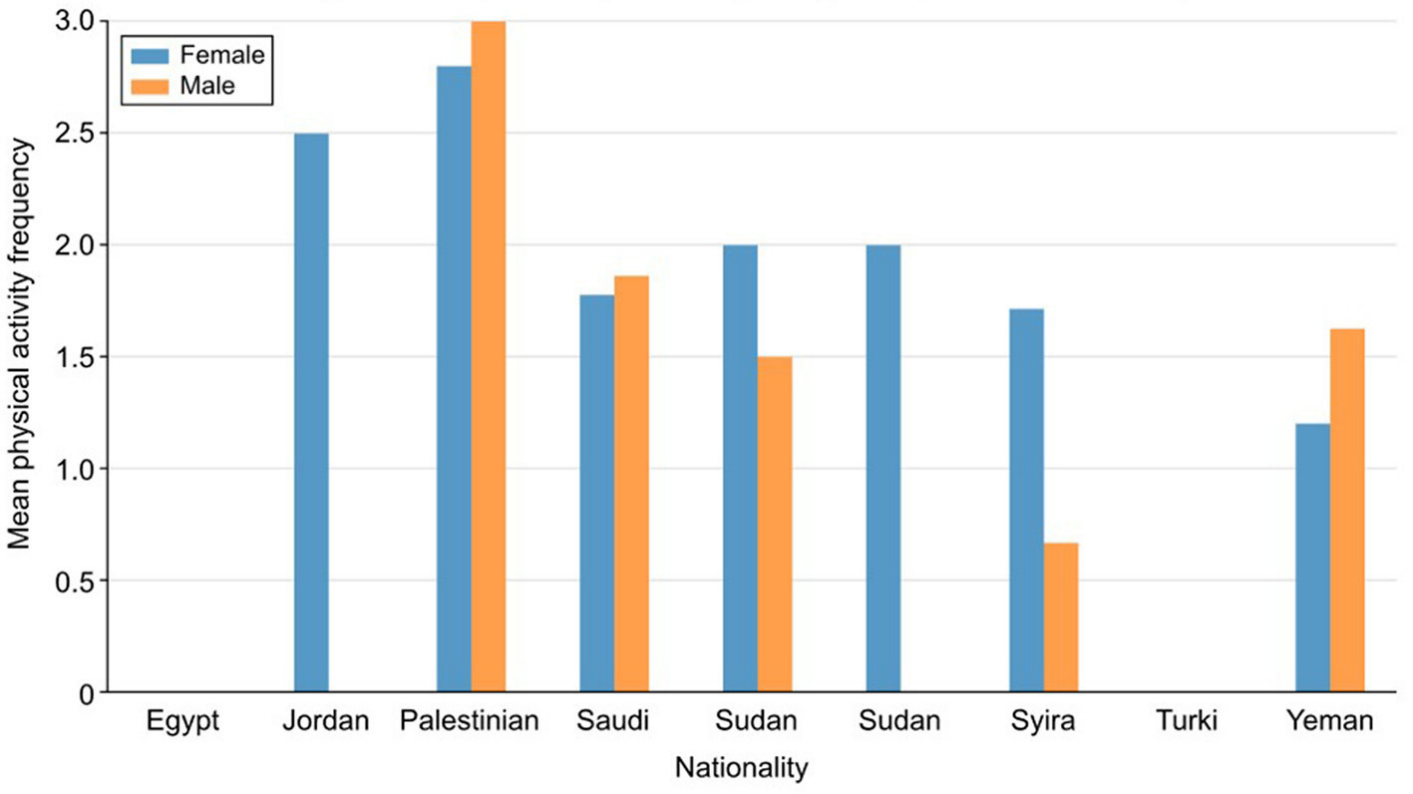
Physical activity by nationality and gender (Saudi vs. non-Saudi participants).

The study highlights significant differences in physical activity levels between Saudi and non-Saudi adolescents. Overall, non-Saudi participants reported lower levels of physical activity and higher safety concerns compared to their Saudi counterparts. For instance, 54% of non-Saudi participants reported “never” participating in indoor recreational activities such as gym sessions or martial arts, whereas 24% of Saudi adolescents reported similar inactivity levels, as shown in Figure 5.

In terms of outdoor activities, non-Saudi participants also exhibited lower engagement. For example, 44% of non-Saudi participants reported “never” participating in desert activities, compared to only 30% of Saudi adolescents. This difference suggests that cultural and environmental factors may play a role in shaping how adolescents from different backgrounds perceive and engage in physical activities.

Safety concerns were more prevalent among non-Saudi participants, further limiting their participation in outdoor activities. Many non-Saudi participants expressed concerns about traffic hazards, poor visibility, and the lack of pedestrian infrastructure in their neighborhoods. For instance, 40% of non-Saudi participants strongly disagreed that the traffic in their neighborhood was slow enough for safe walking, compared to 33% of Saudi participants. This indicates that non-Saudi adolescents face additional barriers to physical activity due to a heightened perception of safety risks, which may be linked to their residential environment or socio-economic status.

These findings suggest that cultural and national backgrounds significantly influence physical activity patterns, with non-Saudi adolescents being more susceptible to inactivity due to both environmental and safety-related factors. Addressing these concerns through targeted interventions, such as improving neighborhood safety and accessibility to recreational facilities, could help encourage higher participation in physical activity among non-Saudi young people.

### 4.5 Result correlations

The results of the study reveal several important correlations that highlight how various factors influence physical activity levels among adolescents. These correlations demonstrate the interconnectedness between environmental, socio-economic, and personal perceptions of safety and the frequency of physical activity. By examining relationships such as the impact of traffic hazards, pollution, and neighborhood safety perceptions, as well as the role of gender, community involvement, and socio-economic status, the findings provide a deeper understanding of barriers to, and facilitators of, physical activity. These correlations underscore the need for targeted interventions to improve environmental conditions, enhance safety, and increase community engagement to promote healthier lifestyles among young people.

#### 4.5.1 Traffic hazards and physical activity

Table 3 highlights the correlation between perceived traffic hazards and the frequency of outdoor physical activities such as walking and biking. Adolescents who perceived higher traffic hazards were less likely to participate in these activities. For example, 40% of those perceiving high traffic hazards reported never walking outdoors, compared to only 15% of those perceiving low traffic hazards. Similarly, 45% of those who perceived high traffic hazards reported never biking, in contrast to only 20% of those perceiving low traffic hazards. These results emphasize the impact of traffic safety on adolescents' outdoor physical activity levels, suggesting that better traffic management and infrastructure improvements could encourage more frequent participation in outdoor activities.

**Table 3:** Relation between perceived traffic hazards vs. frequency of outdoor physical activities.

**Perception of traffic hazards**	**Frequency of outdoor activity**	**Never (%)**	**Once a month or less (%)**	**2–3 times/week (%)**	**4+ times/week (%)**
High traffic hazard	Walking	40%	35%	15%	10%
Low traffic hazard	Walking	15%	25%	30%	30%
High traffic hazard	Biking	45%	30%	15%	10%
Low traffic hazard	Biking	20%	30%	25%	25%

#### 4.5.2 Safety perceptions and activity frequency

Table 4 presents the correlation between neighborhood safety perceptions (visibility and crosswalk safety) and outdoor activity levels. Adolescents who reported poor visibility in their neighborhoods were less likely to walk, with 35% never walking, compared to only 10% never walking in areas with good visibility. Similarly, 45% of adolescents who perceived crosswalks as unsafe reported never engaging in outdoor recreational activities, while only 15% of those in neighborhoods with safe crosswalks reported the same. This table indicates that improvements in visibility and pedestrian infrastructure can significantly influence the frequency of outdoor physical activity.

**Table 4:** Correlation between neighborhood safety perceptions and outdoor activity levels.

**Neighborhood safety perception**	**Activity type**	**Never (%)**	**Once a month or less (%)**	**2–3 times/week (%)**	**4+ times/week (%)**
Poor visibility	Walking	35%	40%	15%	10%
Good visibility	Walking	10%	30%	30%	30%
Unsafe crosswalks	Outdoor recreation	45%	35%	15%	5%
Safe crosswalks	Outdoor recreation	15%	25%	35%	25%

#### 4.5.3 Pollution and outdoor activity

Table 5 illustrates how concerns about pollution, particularly exhaust fumes, correlate with outdoor physical activity. In neighborhoods with high pollution levels, 50% of adolescents reported never walking outdoors, while only 15% of adolescents in low-pollution areas reported similar inactivity. The pattern is similar for biking activities, where 45% of adolescents in high-pollution areas never participated in biking compared to only 20% in low-pollution areas. These findings suggest that environmental pollution is a significant deterrent from outdoor physical activity, particularly in urban areas.

**Table 5:** How concerns about pollution, particularly exhaust fumes, correlate with outdoor physical activity.

**Perception of pollution**	**Outdoor activity**	**Never (%)**	**Once a month or less (%)**	**2–3 times/week (%)**	**4+ times/week (%)**
High pollution	Walking	50%	30%	15%	5%
Low pollution	Walking	15%	25%	35%	25%
High pollution	Biking	45%	35%	15%	5%
Low pollution	Biking	20%	30%	30%	20%

## 5 Discussion

The findings of this study provide a nuanced understanding of the interplay between neighborhood safety, environmental factors, and socio-cultural influences in relation to adolescent physical activity levels in Saudi Arabia. The results highlight several critical determinants, including traffic safety, crime, pollution, proximity to recreational spaces, and socio-economic disparities, all of which shape physical activity patterns among adolescents. Notably, adolescents who perceived higher traffic-related safety concerns reported 30% lower physical activity levels compared to those who felt that their neighborhoods were safer (*p* < 0.01), and female adolescents were 20% more likely to report safety concerns than their male peers (*p* < 0.05). These results indicate that gender-specific barriers, socio-economic status, and access to safe outdoor spaces are significant factors affecting adolescent physical activity in the region.

It is relevant to point out the role of cultural norms, especially in conservative countries like Saudi Arabia, where gender-related barriers make a great impact on the level of physical activities. The expectations placed by society and the limitation of public behavior impede the chances of outdoor activities among young females. Future research in these cultural dynamics will help in the design of culturally sensitive interventions that respect societal norms while promoting physical activity, such as culturally appropriate female-only facilities or programs that promote family-based activities to create more inclusive environments.

### 5.1 Neighborhood safety and gender-specific barriers

Traffic safety concerns and perceptions of crime emerged as significant barriers, particularly among female adolescents. Cultural norms in Saudi Arabia further restrict girls' participation in outdoor activities, with girls expressing heightened sensitivity to neighborhood safety issues such as traffic volume and visibility. For example, 558 participants (mostly girls) strongly disagreed that walkers and bikers could be easily seen by people in their homes. As a result, girls reported engaging in 25% less physical activity compared to boys, primarily due to safety-related barriers. These findings suggest that creating female-only recreational spaces, such as gyms and parks, or offering women-only hours at public facilities, could encourage more physical activity among girls. In Saudi Arabia, cultural norms emphasize gender segregation in public spaces to ensure privacy and comfort for women. Providing dedicated spaces for females aligns with these cultural expectations and could help overcome barriers that limit girls' participation in physical activities. Additionally, the development of safe walking and biking routes to schools and parks, along with better street lighting, could help mitigate safety concerns.

The perception of neighborhood safety and subsequent levels of physical activity among adolescents are highly influenced by Saudi cultural values. Traditional gender roles usually place more restrictions on girls, making them less likely to access public spaces and thus more sensitive to safety factors such as visibility and traffic. These cultural expectations further influence parental decisions, with girls often having fewer opportunities for outdoor activities compared to boys. These culturally entrenched barriers will be addressed only with gender-specific interventions, such as provisions for female-only recreational space, and safety initiatives framed in a culturally congruent manner that encourage equitable participation in physical activities.

The recommendation to create women's-only recreational spaces is in harmony with Saudi Arabia's cultural concerns for gender separation in public. These could be included in current community facilities in such a way that accessibility and privacy are guaranteed. These programs should always promote inclusivity by offering complementary activities in mixed-gender environments—for example, family-oriented parks, or events that allow members of the community to take part—so as not to socially exclude someone. Interventions will need to be individually designed by policymakers in harmony with local stakeholders and their community leaders, and should effectively balance safety concerns with support for social integration on a broader scale.

### 5.2 Impact of residential environment and socio-economic disparities

Adolescents living in suburban settings with access to private yards or nearby parks were more physically active, reporting 15% higher activity levels compared to their peers in urban areas. In contrast, those residing in flats expressed greater concern over traffic safety and pollution, contributing to lower engagement in outdoor activities. For instance, 720 participants (48%) strongly disagreed that their neighborhood was free from exhaust fumes, and those from lower-income areas reported limited access to recreational facilities. These disparities emphasize the need for equitable distribution of recreational spaces and safe pedestrian infrastructure, particularly in lower-income neighborhoods. Policymakers should prioritize the development of public parks, green spaces, and sports facilities within urban areas to promote physical activity among adolescents.

Socio-economic inequalities significantly shape both physical activity levels and perceptions of safety. Adolescents in lower-income neighborhoods often face limited access to safe recreational spaces, inadequate infrastructure, and higher exposure to environmental hazards, which discourage physical activity. To address these disparities, targeted policies should focus on equitable distribution of resources, such as creating well-maintained parks, improving pedestrian infrastructure, and subsidizing community programs in underprivileged areas. Empowering local communities through participatory urban planning can also ensure that interventions effectively meet the needs of marginalized populations.

### 5.3 Proximity to recreational areas and community engagement

The proximity of recreational areas, such as parks and sports facilities, significantly influenced physical activity levels. Adolescents with easy access to these spaces were more likely to engage in regular outdoor activities, while those living farther away reported lower participation. This was especially true for those in urban areas, where limited access to parks was associated with reduced physical activity. Additionally, community engagement played a critical role in promoting active lifestyles. Adolescents involved in community organizations, such as sports clubs or mosques, reported engaging in physical activities more frequently. This suggests that community-driven interventions and the establishment of structured programs in community settings can help increase physical activity levels, particularly in underserved areas.

### 5.4 Cultural and national differences

Cultural and national backgrounds were found to influence physical activity levels, with non-Saudi adolescents reporting lower engagement in outdoor activities and higher safety concerns compared to their Saudi peers. For instance, 54% of non-Saudi participants reported never participating in indoor recreational activities, such as gym sessions or martial arts, compared to 24% of Saudi adolescents. Safety concerns were also more prevalent among non-Saudi respondents, limiting their participation in outdoor activities. Addressing these cultural differences through targeted interventions and community engagement can help reduce disparities and promote higher levels of physical activity across diverse adolescent populations.

The observed discrepancies in physical activity levels and safety perceptions between Saudi and non-Saudi residents likely stem from sociocultural differences and varying levels of integration within the local context. Non-Saudi adolescents may experience heightened safety concerns due to unfamiliarity with local norms, limited access to community networks, and differences in neighborhood infrastructure. These challenges are exacerbated by socio-economic disparities, as non-Saudi families often reside in less affluent areas with fewer recreational opportunities. Future research should delve deeper into these sociocultural dynamics and consider including targeted interventions that address integration barriers and promote inclusive community engagement. Relevant studies, such as those by Zeng et al. (32) and Constable Fernandez et al. (4), provide additional context for understanding these dynamics.

Non-Saudi participants face unique challenges due to socio-economic disparities, limited integration into local communities, and cultural differences. These factors may often exacerbate perceptions of neighborhood safety and reduce opportunities for physical activity. Targeted interventions that address these challenges should focus on promoting cultural integration, improving recreational facilities in less affluent areas, and developing community-wide programs inclusive of all adolescents, regardless of nationality, to engage in physical activity.

### 5.5 Environmental pollution and health risks

Pollution, particularly air pollution from traffic and industrial sources, was identified as a major deterrent from outdoor physical activity, especially in urban areas. Adolescents living in densely populated urban environments reported that exposure to pollution, such as exhaust fumes, discouraged them from engaging in activities such as walking, biking, or playing sports. This reluctance contributes to a sedentary lifestyle and increases the risk of health problems such as obesity and cardiovascular diseases. Urban planning strategies that incorporate green spaces and create pollution-free recreational areas are crucial in promoting outdoor activities and reducing health risks. For instance, creating superblocks or pedestrian-only streets in residential areas could help minimize exposure to traffic-related pollution and encourage more physical activity.

While this study identified pollution as a major environmental barrier to physical activity, other environmental factors such as the availability of green space, urban density, and climate conditions are important in determining the level of activity. This broader analysis of these elements may be included in further studies to provide a comprehensive understanding of how the physical environment shapes adolescent physical activity patterns. Such an approach would enable more focused urban planning interventions aimed at addressing environmental barriers holistically.

### 5.6 Implications for urban planning and policy

The built environment plays a critical role in shaping adolescent physical activity levels. Well-designed neighborhoods with safe pedestrian infrastructure, such as sidewalks, bike lanes, and crosswalks, create environments where physical activity is more accessible and appealing. In contrast, car-centric urban designs and limited access to green spaces discourage active behaviors. To promote healthier lifestyles, urban planners and policymakers should prioritize pedestrian-friendly environments, implement safe route-to-school programs, and ensure the equitable distribution of parks and recreational facilities. Additionally, incorporating public health goals into urban design, such as reducing pollution and promoting active commuting, can create environments that support physical activity and improve long-term health outcomes for adolescents.

### 5.7 Recommendations for future research and policy interventions

Future research should focus on longitudinal studies to explore how neighborhood safety perceptions and environmental factors influence physical activity levels over time. Integrating objective measures of physical activity, such as wearable devices, could provide more accurate data and reduce self-report bias. There is also a need for gender-inclusive interventions that address cultural constraints and promote physical activity among girls in conservative societies. Policymakers should consider implementing community-driven initiatives that leverage existing social networks to increase physical activity opportunities for adolescents. Urban planning strategies that integrate green spaces and create safer pedestrian environments can further support active lifestyles, particularly in rapidly urbanizing regions such as Saudi Arabia.

The findings of this study resonate with global research emphasizing the critical role of neighborhood safety in promoting adolescent physical activity. For instance, studies in South Africa (5) and the United States (18) have similarly identified traffic safety and environmental hazards as significant barriers. Comparing these results with Saudi Arabia highlights universal challenges, such as traffic management and the need for safe recreational spaces, while also underscoring region-specific factors like cultural norms. These parallels suggest that international collaboration on urban planning and public health strategies can provide valuable insights to address both global and local barriers to adolescent physical activity.

A longitudinal study design might have a better chance of revealing the dynamic relationship existing between perceived neighborhood safety and physical activity level. Changes over time would allow identification of the cause and long-term impact of safety interventions. This approach might also consider developmental changes in adolescents and dynamic environmental conditions and provide a broader perspective that is necessary to inform policy and urban planning based on evidence.

Urban planners and public health authorities should implement specific measures such as developing pedestrian-friendly infrastructure, increasing green space access, and introducing traffic-calming measures in high-risk areas. Additionally, community-based programs that engage residents in promoting safety and activity-friendly environments can ensure long-term sustainability. Policymakers should prioritize equitable resource allocation to underserved neighborhoods to address disparities identified in this study.

Overall, this study underscores the importance of neighborhood safety, residential environment, and socio-economic status in shaping adolescent physical activity patterns. Addressing these factors through targeted policy interventions and community engagement can help promote healthier lifestyles and reduce disparities in physical activity levels among adolescents. By improving access to safe, inclusive environments and creating opportunities for structured physical activities, urban planners and policymakers can support the long-term health and wellbeing of future generations.

## 6 Conclusions

The study provides a comprehensive understanding of how neighborhood safety, environmental factors, and socio-economic disparities influence adolescent physical activity levels in Saudi Arabia's rapidly urbanizing regions. The results reveal that perceptions of neighborhood safety, particularly related to traffic hazards, visibility, and crime, play a significant role in determining adolescents' engagement in outdoor activities such as walking, biking, and sports. Adolescents who perceived traffic safety as a concern engaged in 30% less physical activity compared to those in safer neighborhoods, with female adolescents being 2.3 times more likely to report such concerns, contributing to a 20% reduction in outdoor activity among girls (*p* < 0.05). Additionally, the residential environment significantly influenced activity levels, with adolescents in suburban settings reporting 15% higher outdoor activity participation than those living in urban flats (*p* < 0.05). Pollution concerns were also more prevalent in urban areas, where 720 participants (48%) strongly disagreed with the statement that their neighborhood was free from exhaust fumes, and this issue further deterred outdoor activities. Socio-economic disparities were evident, as adolescents in lower-income settings, such as flats, reported higher inactivity levels and greater safety concerns compared to those living in more affluent environments such as villas. For instance, 166 out of 558 adolescents in flats reported never participating in indoor recreational activities compared to only 52 out of 274 villa residents. Gender-specific barriers were pronounced, with girls expressing higher safety concerns, such as inadequate visibility and unsafe traffic conditions, leading to a 25% lower physical activity level compared to boys. Community engagement emerged as a positive factor, as adolescents involved in three or more community organizations engaged in physical activities more frequently, whereas 41% of those not engaged in any organizations reported rarely participating in physical activities. These findings highlight the need for targeted interventions, such as traffic-calming measures, improved street lighting, the development of well-maintained green spaces, and addressing pollution concerns to promote higher physical activity levels among adolescents, particularly girls and those in urban and lower-income settings. By improving neighborhood safety and creating safer, more inclusive environments, policymakers and urban planners can help adolescents lead more active and healthier lives, ensuring better long-term health outcomes.

## Data Availability

The original contributions presented in the study are included in the article/supplementary material, further inquiries can be directed to the corresponding author.
